# Upward elevation and northwest range shifts for alpine *Meconopsis* species in the Himalaya–Hengduan Mountains region

**DOI:** 10.1002/ece3.5034

**Published:** 2019-03-11

**Authors:** Xie He, Kevin S. Burgess, Xue‐Fei Yang, Antje Ahrends, Lian‐Ming Gao, De‐Zhu Li

**Affiliations:** ^1^ Germplasm Bank of Wild Species Kunming Institute of Botany, Chinese Academy of Sciences Kunming Yunnan China; ^2^ CAS Key Laboratory for Plant Diversity and Biogeography of East Asia, Kunming Institute of Botany Chinese Academy of Sciences Kunming Yunnan China; ^3^ Kunming College of Life Science University of Chinese Academy of Sciences Kunming Yunnan China; ^4^ Department of Biology, College of Letters and Sciences Columbus State University, University System of Georgia Columbus Georgia; ^5^ Key Laboratory of Economic Plants and Biotechnology Kunming Institute of Botany, Chinese Academy of Sciences Kunming Yunnan China; ^6^ Royal Botanic Garden Edinburgh Edinburgh UK

**Keywords:** Alpine ecosystems, biodiversity hotspots, global climate change, *Meconopsis*, range shift

## Abstract

Climate change may impact the distribution of species by shifting their ranges to higher elevations or higher latitudes. The impacts on alpine plant species may be particularly profound due to a potential lack of availability of future suitable habitat. To identify how alpine species have responded to climate change during the past century as well as to predict how they may react to possible global climate change scenarios in the future, we investigate the climatic responses of seven species of *Meconopsis*, a representative genus endemic in the alpine meadow and subnival region of the Himalaya–Hengduan Mountains. We analyzed past elevational shifts, as well as projected shifts in longitude, latitude, elevation, and range size using historical specimen records and species distribution modeling under optimistic (RCP 4.5) and pessimistic (RCP 8.5) scenarios across three general circulation models for 2070. Our results indicate that across all seven species, there has been an upward shift in mean elevation of 302.3 m between the pre‐1970s (1922–1969) and the post‐1970s (1970–2016). The model predictions suggest that the future suitable climate space will continue to shift upwards in elevation (as well as northwards and westwards) by 2070. While for most of the analyzed species, the area of suitable climate space is predicted to expand under the optimistic emission scenario, the area contracts, or, at best, shows little change under the pessimistic scenario. Species such as *M. punicea*, which already occupy high latitudes, are consistently predicted to experience a contraction of suitable climate space across all the models by 2070 and may consequently deserve particular attention by conservation strategies. Collectively, our results suggest that the alpine high‐latitude species analyzed here have already been significantly impacted by climate change and that these trends may continue over the coming decades.

## 1 INTRODUCTION

Species may respond to climate change by shifting their ecological niche through plastic changes (Nicotra et al., 2010) and evolutionary adaptation (Visser, 2008), and/or by shifting their range to track original climatic conditions (Hickling, Roy, Hill, Fox, & Thomas, 2006; Holt, 1990). Evidence suggests that the rate of species climatic niche evolution may be slow compared to the rate of climate change (Quintero & Wiens, 2013), and failure to respond to the changing abiotic and biotic conditions may lead to range contractions (Giménez‐Benavides, Albert, Iriondo, & Escudero, 2011) and/or local extinctions (Moritz & Agudo, 2013; Wiens, 2016).

In plants, the most commonly documented responses to climate change are changes in phenology (Cleland, Chuine, Menzel, Mooney, & Schwartz, 2007) and distributional range shifts to higher latitudes and/or elevations (Chen, Hill, Ohlemuller, Roy, & Thomas, 2011; Parmesan & Yohe, 2003). Range shifts are thus an important climate change “coping” strategy, and they have been documented in a wide range of studies, including global‐scale meta‐analyses (Lenoir, Gegout, Marquet, de Ruffray, & Brisse, 2008; Wiens, 2016), plot monitoring (Keller, Kienast, & Beniston, 2000; Pauli, Gottfried, & Grabherr, 2003), resurveys of plots (Kelly & Goulden, 2008; Morueta‐Holme et al., 2015), and analyses of historical specimen records (Feeley, 2012; Wolf, Zimmerman, Anderegg, Busby, & Christensen, 2016). While range shifts may contribute to species’ survival, they may also expose them to new biotic and abiotic pressures they are maladapted to, lead to a breakdown of species interactions, and threaten the stability of existing communities and local endemism (Harley, 2011; Kharouba & Vellend, 2015). Declines in reproductive success (Galloway & Burgess, 2012), lowered species abundance (Calinger, 2015), reduced adaptive variation/intraspecific genetic diversity (Pauls, Nowak, Balint, & Pfenninger, 2013), changes in traits associated with mating systems (Etterson & Mazer, 2016), and the encroachment of invasive species (Beans, Kilkenny, & Galloway, 2012; Bellard et al., 2013) are some of many potential consequences. Given the potentially profound ecological implications, and the fact that range shifts have accelerated over the last decades (Steinbauer et al., 2018; Walther, Sascha, & Burga, 2005), documenting the patterns and magnitude is thus important for understanding species and community persistence.

Due to their restricted distribution range and high levels of endemism, alpine species, in particular, are generally highly sensitive to climate change (Jump, Huang, & Chou, 2012; Lenoir et al., 2008). Cold‐adapted species (mainly nival and subnival species) endemic to the summit region of mountain systems tend to decline in abundance or contract in range size (Pauli, Gottfried, Reiter, Klettner, & Grabherr, 2007; Rumpf et al., 2018), while low‐elevation species adapted to warmer temperatures encroach. For example, numerous studies in Europe have documented how species adapted to warmer temperatures have occupied habitats previously occupied by the cryophilous subnival flora, which, as a result of increased competition for cooler habitats and limited space for new habitat expansion, is then restricted to small patches “trapped” in remaining habitats where cooler conditions persist (Gottfried et al., 2012; Gottfried, Pauli, Reiter, & Grabherr, 1999; Pauli et al., 2003). However, the extent of distributional range shifts due to climatic change for alpine species remains poorly understood.

The Himalaya–Hengduan Mountains region is located in a global biodiversity hotspot which, due to its recent geological history and diversity of habitats, supports alpine regions containing relatively high levels of nival and subnival plant diversity and endemism (Xu, Li, & Sun, 2014). Recent photographic comparisons have shown that climate change during the past several decades has caused glacier retreat and subsequent upward shifts of the alpine tree line in the area (Baker & Moseley, 2007). This is in line with findings for the wider Asian mountain region, where there is widespread evidence for climate‐related glacier shrinkage and tree and shrub line advancement (Cogley, 2016; Du et al., 2018; Myers‐Smith & Hik, 2017), potentially threatening regional endemism in alpine communities.

**Figure 1:**
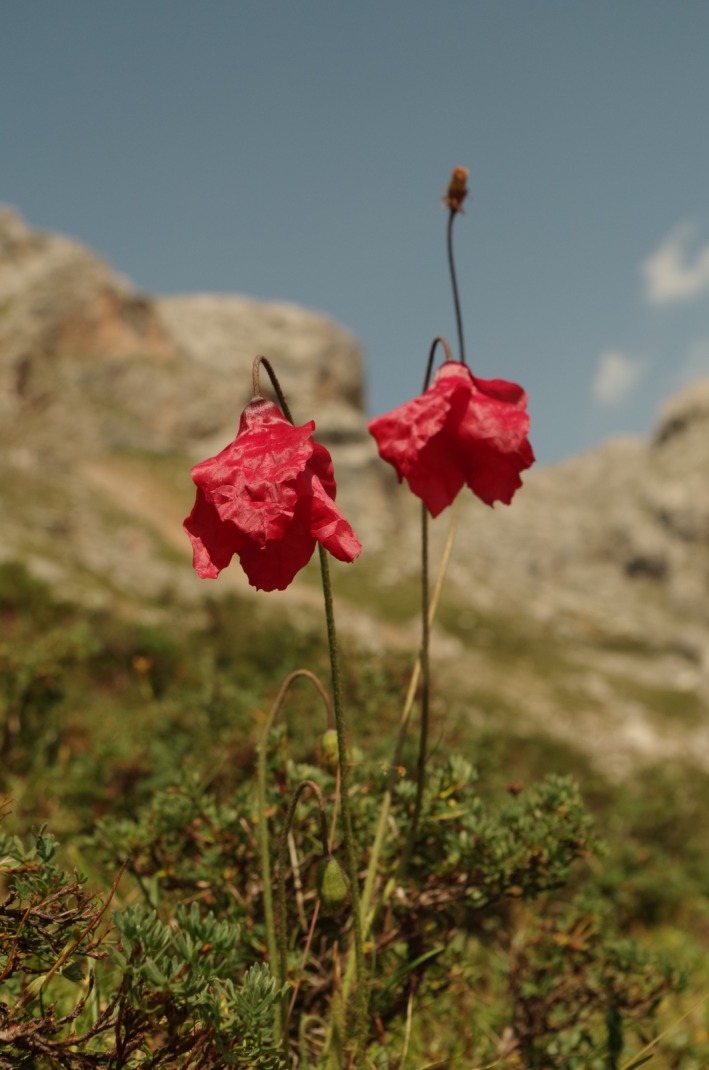
One of the species studied: *Meconopsis punicea*, photographed in Gansu province, China (2016)

*Meconopsis*, commonly known as Himalayan blue poppies, is a genus of the Papaveraceae with ~60 species confined to alpine meadow or subnival habitats (Figure 1) in the Himalaya–Hengduan Mountains region. It is verified by recent molecular phylogenies (Liu, Liu, Yang, & Wang, 2014; Xiao & Simpson, 2017) with a conserved type, *M. regia* (Grey‐Wilson, 2012). *Meconopsis* species are entomophilous plants, and they mainly attract flies as pollinators by providing them with a warm shelter (Wu et al., 2015). Due to their restricted ranges and limited pollinators in high‐elevation habitats, species of *Meconopsis* may be particularly sensitive to climate change and are an ideal model to investigate the climatic responses of plants in this biodiversity hotspot. Here, we investigate the climatic responses of seven species of *Meconopsis* during the past century to predict how they may react to possible global climate change in the coming decades. We use specimen records over the past one hundred years to see whether significant historical shifts in elevation have occurred. To explore potential future shifts in longitude, latitude, elevation, and range size, we used a species distribution modeling (SDM) framework to project the future distributions of the species under optimistic and pessimistic greenhouse gas scenarios in 2070. In addition, we compared historical rates of shifts in elevation with future projections to evaluate the validity of model projections and to evaluate species persistence under climate warming.

## 2 MATERIALS AND METHODS

### 2.1 Occurrence data

Occurrence data were obtained from three sources: (a) specimen records from the Chinese Virtual Herbarium (CVH: http://www.cvh.ac.cn/), the Specimen Resources Sharing Platform for Education (SRSPE: http://mnh.scu.edu.cn/main.aspx), and the Global Biodiversity Information Facility (GBIF: https://www.gbif.org/); (b) seed collection information from the Germplasm Bank of Wild Species of CAS’ Kunming Institute of Botany (GBOWS: http://www.genobank.org/); (c) distribution information of *Meconopsis* species in the published literature (Liu et al., 2014; Shang et al., 2015; Yang, Qin, Li, & Wang, 2012; Yang et al., 2010) and from field collections of our colleagues in the last decade.

We first collected the distribution information of all the species of *Meconopsis* that had occurrence data in the Himalaya–Hengduan Mountains region (3,745 samples for 35 species). Seven species of *Meconopsis* that had a representative number of specimens ranging between 147 and 807 (*N* = 2,911; Supporting information Table S1) were included in our analysis to ensure that we would have enough valid data for subsequent analyses. We removed duplicated specimens with the same collection number, specimens with problematic identification and/or potentially erroneous locality information, and specimens without collection year.

**Figure 2:**
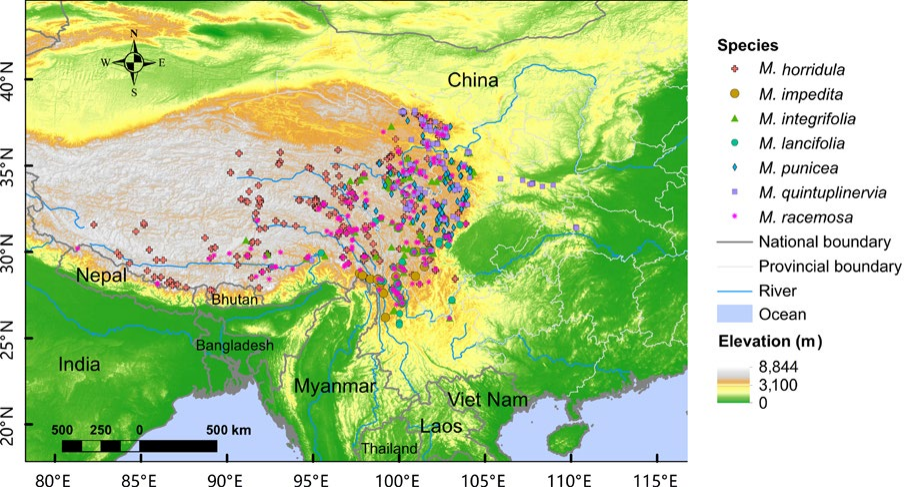
Occurrence of the seven species of *Meconopsis* in the Himalaya–Hengduan Mountains (*N* = 793 for all the seven species)

Two sets of data were generated respectively following different criteria. (a) For data used in SDM, we restricted our analysis to specimens collected after 1950 that had a detailed description of their collection localities, which were then used to search on Google Earth for precise spatial coordinates. We combined occurrence data from specimens, field collections, and the published literature and removed duplicate occurrences within a 5‐kilometer range (yielding a total of *N* = 793 records with numbers of records per species ranging from 19 to 252, Figure 2; Supporting information Table S1) in order to lower the potential autocorrelation through spatial filtering. (b) For analysis of historical shifts in elevation, only those specimens with detailed elevation and collection date (year) were used. With data from field collections and published literature combined, 2,541 records remained spanning from year 1922 to 2016 (Table 1; Supporting information Figure S1).

**Table 1:** Mean elevation (m ± standard error) of the seven species of *Meconopsis* species in the Himalaya–Hengduan Mountains

Species	Pre‐1970s		Post‐1970s		*p*‐Value	Shifting rates (m/per decade)
	Occurrence number	Elevation	Occurrence number	Elevation		
*M. horridula*	104	4,406.4 ± 59.6	421	4,476.1 ± 23.6	0.279	21.1
*M. impedita*	54	3,947.8 ± 63.2	29	4,297.9 ± 59.6	0.0001328***	68.9
*M. integrifolia*	264	3,721.4 ± 28.9	477	4,105.6 ± 21.5	<2.2e−16***	93.4
*M. lancifolia*	49	3,905.1 ± 49.8	54	4,118.9 ± 65.8	0.0111*	47.8
*M. punicea*	76	3,702.2 ± 59.9	168	3,925.6 ± 38.8	0.002121**	59.1
*M. quintuplinervia*	110	3,427.0 ± 46.9	200	3,585.7 ± 36.9	0.008384**	43.6
*M. racemosa*	217	3,875.8 ± 34.7	318	4,140.3 ± 26.0	2.244e−09***	65.0

Notes

Elevational records are listed for the two time periods (pre‐1970s: 1922–1969; post‐1970s: 1970–2016) in this study.

^#^Significant historical shifts in elevation between the two time periods. *p < 0.05, **p < 0.01,***p <0.001.

### 2.2 Historical shifts in elevation

We divided the specimens with elevational records into two equal time periods of c. 50 years each (pre‐1970s: 1922–1969 and post‐1970s: 1970–2016). This split reflects a turning point in global (and local Himalayan) temperature trends, which exhibit a steady increase since 1970 (IPCC, 2014; Shrestha, Wake, Mayewski, & Dibb, 1999). It should be noted that due to the vacancy of refined tools to estimate the precise elevation in the early years, the pre‐1970s elevational records normally have an approximate accuracy of 50 or 100 m compared to post‐1970s data, which in contrast have much finer and precise values. In total, there were 874 elevational records for the pre‐1970s and 1667 for the post‐1970s, and the mean year of occurrences was 1950 and 1990, respectively. The number of elevational records per species and time period ranged from a minimum of 29 to a maximum of 477 (Table 1). Shifts in mean elevation within and between time periods were then compared for each species separately as well as across all species collectively, and significance was established using paired *t* tests.

### 2.3 Species distribution modeling

To project the species distributions to different climate change scenarios in 2070, bioclimatic variables of current conditions (Current: the average for 1960–1990) and future conditions (2070: the average for 2060–2080) were downloaded from WorldClim (http://www.worldclim.org/) at the highest available spatial resolution (30 arc‐seconds; ~1 km). We used three global circulation models (GCM)—ACCESS1‐0, BCC‐CSM1‐1, and HadGEM2‐ES (hereafter abbreviated as AC, BC, and HE), each combined with two greenhouse gas concentration trajectories: an optimistic scenario whereby emissions peak around 2040 and then decline (Representative Concentration Pathway (RCP 4.5), and a pessimistic scenario whereby emissions continue to rise throughout the century (RCP 8.5). Thus, there were six potential future scenarios in total. The three GCMs were chosen as they were evaluated to perform best in terms of both temperature and precipitation in Himalaya–Hengduan Mountains region (Wu, Jiang, & Xie, 2017; Zhang, Zhang, & Fan, 2015). A Pearson's correlation test was implemented for each pair of the 19 climatic variables downloaded (Supporting information Table S2), and we removed the highly correlated variables with correlation coefficients above 0.90. After this procedure, 8 bioclimatic variables, including four variables associated with temperature (bio1: annual mean temperature; bio2: mean diurnal range; bio3: isothermality; and bio4: temperature seasonality) and four variables associated with precipitation (bio12: annual precipitation; bio14: precipitation of driest month; bio15: precipitation seasonality; and bio18: precipitation of warmest quarter), were used in our analyses. All the layers were cut and standardized to the same resolution (30 arc‐seconds) using a mask fitted to the species distribution region (78°E–117°E, 22°N–45°N) with the same coordinate system (WGS 1984) and transferred to ASCII format to enable the operation in the model.

Our species distribution models were based on Maximum Entropy Modeling (MaxEnt), which has been shown to be the most appropriate technique for modeling presence‐only data (Elith et al., 2011; Phillips, Anderson, & Schapire, 2006). We set the regularization multiplier value as “2” to reduce overfitting (Radosavljevic, Anderson, & Araújo, 2014) and the maximum iterations as “1,000” to allow more time for convergence. We used the average output (based on ten replicate cross‐validation runs for each species) for subsequent analyses. We reclassified the MaxEnt output file using the 10‐percentile training presence logistic threshold value to define a species potential distribution region, above which species were considered “present” in the region, a method widely recognized for distinguishing suitable from unsuitable regions (Deb, Phinn, Butt, & McAlpine, 2017; Hughes, 2017; Kramer‐Schadt et al., 2013; Radosavljevic et al., 2014). We then calculated the longitude, latitude, elevation, and range size of each cell of potential presence, compared the average value of longitude, latitude, and elevation between the current time period and 2070, and the predicted shifts in total range size for 2070.

All the analyses were performed using MaxEnt 3.3.3k (Phillips et al., 2006), R x64 3.3.3 (R Core Team, 2016), and ArcGIS 10.2 (Environmental Systems Resource Institute, 2014).

## 3 RESULTS

### 3.1 Historical shifts in elevation

Across the seven *Meconopsis* species, there was a significant shift in mean elevation of 302.3 m (*t* = −13.004, *df* = 1737.8, *p*‐value <2.2e−16) between the two time windows. Records collected pre‐1970s had a mean elevation of 3,826.8 m (±561.0 m) and covered a range of 2,000.0–5,600.0 m. Records collected post‐1970s had a mean elevation of 4,129.1 m (±548.2 m) and ranged from 2,289.0 to 5,559.0 m (Supporting information Figure S2a). When analyzed separately, each species showed upward shifts in mean elevation between the two time periods: Elevational shifts ranged from 69.7 m (*M. horridula*) to 384.3 m (*M. integrifolia*), and the elevational shifts in six out of the seven species were significantly different between the two time periods. The shifting rates range from 21.1 m (*M. horridula*) to 93.3 m per decade (*M. integrifolia*), and the average rate for all the species is 56.9 m per decade (Table 1; Supporting information Figure S2b).

### 3.2 Projected distributions of species

**Figure 3:**
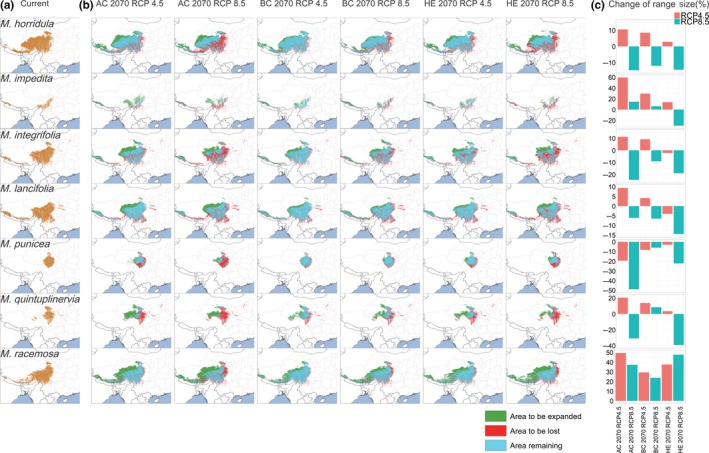
Comparison of (a) the predicted distribution region for each species in the current time period; (b) and the overlay of the current time period and the year 2070 for ACCESS1‐0 (AC), BCC‐CSM1‐1 (BC), and HadGEM2‐ES (HE) models under RCP 4.5 and RCP 8.5 scenarios, with green region showing the new area to be colonized, blue region showing the area remained still, and red region showing the area to be lost; (c) changes of range size (%) for every species’ six models

**Figure 4:**
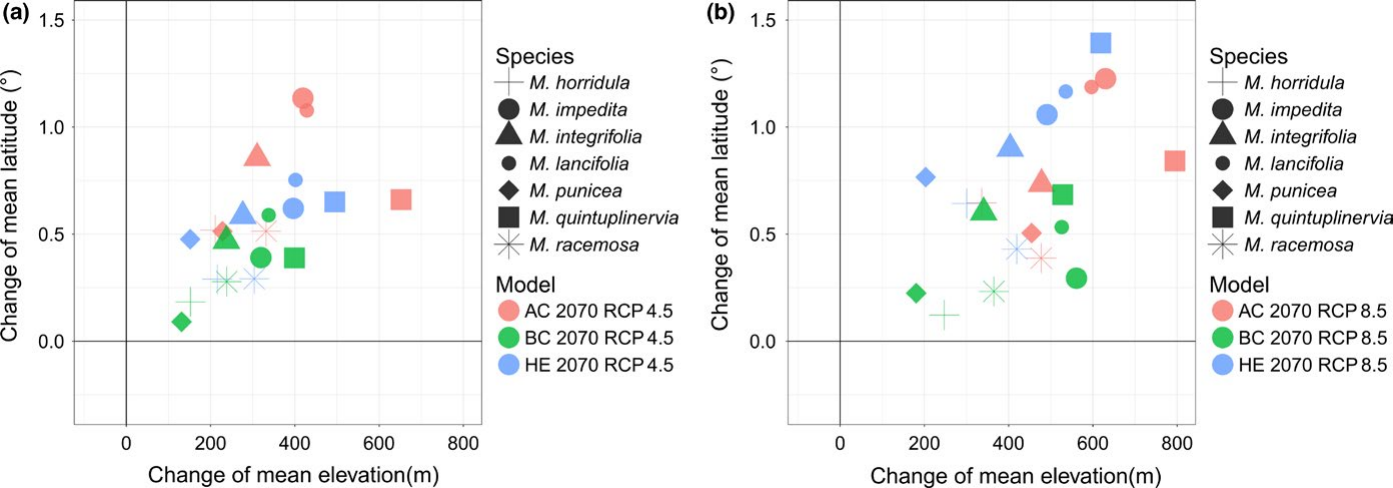
Changes in mean elevation and mean latitude for every species under (a) RCP 4.5 and (b) RCP 8.5 scenarios, with models displayed in different colors and species in different shapes

Projections of current climate preferences onto six climate scenarios for 2070 (three GCMs combined with two RCPs) showed similar trends to the ones established using historical records. For all species and all scenarios, the models suggest that there will be shifts in suitable climate to higher elevations, latitudes, and more westerly longitudes. The magnitude of the predicted shifts varies among the different GCMs and RCPs. For instance, while the BC model predicts a mean shift in suitable habitat (across all seven species) of 0.34° in latitude and 259.34 m in elevation for RCP 4.5, the AC model predicts a mean shift in latitude and elevation of 0.75° and 368.72 m, respectively. For the more pessimistic RCP 8.5 scenario mean model, predictions of shift in suitable habitat range between 0.38° in latitude and 392.67 m in elevation (BC) and 0.79° in latitude and 538.16 m in elevation (AC; Table 2, 3, Figures 3, 4; Supporting information Table S3, S4).

**Table 2:** The RCP 4.5 and RCP 8.5 scenarios of ACCESS1‐0 (AC) model projections for the average distribution in elevation (m ± standard deviation), the range size (km^2^), and the proportion of range size shift (%) between the current time period and the year 2070 for the seven *Meconopsis* species in the Himalaya–Hengduan Mountains

Species	Elevation			Range size (proportion of range size shift)		
	Current	2070 RCP 4.5	2070 RCP 8.5	Current	2070 RCP 4.5 (%)	2070 RCP 8.5 (%)
*M. horridula*	4,461.9 ± 562.2	4,672.8 ± 539.7	4,797.9 ± 469.5	977,160.8	1,079,806.1 (10.5)	832,945.4 (−14.8)
*M. impedita*	4,155.3 ± 554.3	4,574.4 ± 513.2	4,785.3 ± 474.9	133,966.1	213,859.3 (59.6)	153,433.5 (14.5)
*M. integrifolia*	4,102.4 ± 660.4	4,412.8 ± 549.7	4,580.4 ± 480.7	685,546.6	762,052.2 (11.2)	518,117.9 (−24.4)
*M. lancifolia*	3,739.2 ± 936.1	4,167.7 ± 740.0	4,335.9 ± 636.5	764,798.1	836,449.5 (9.4)	718,218.9 (−6.1)
*M. punicea*	3,699.2 ± 544.9	3,927.4 ± 450.4	4,153.8 ± 417.4	224,394.0	181,059.2 (−19.3)	114,493.9 (−49.0)
*M. quintuplinervia*	3,602.3 ± 642.1	4,254.5 ± 477.6	4,397.1 ± 381.5	255,035.0	306,981.6 (20.4)	176,307.8 (−30.9)
*M. racemosa*	4,133.9 ± 557.3	4,465.5 ± 534.9	4,611.1 ± 469.5	679,801.5	1,014,947.0 (49.3)	933,248.3 (37.3)

**Table 3:** The RCP 4.5 and RCP 8.5 scenarios of ACCESS1‐0 (AC) model projections for the average distribution in longitude (°) and latitude (°; ±standard deviation) between the current time period and the year 2070 for the seven *Meconopsis* species in the Himalaya–Hengduan Mountains

Species	Longitude			Latitude		
	Current	2070 RCP 4.5	2070 RCP 8.5	Current	2070 RCP 4.5	2070 RCP 8.5
*M. horridula*	95.8 ± 4.9	93.8 ± 5.7	92.9 ± 5.4	32.0 ± 2.4	32.5 ± 2.5	32.6 ± 2.4
*M. impedita*	96.7 ± 7.5	96.0 ± 7.2	94.6 ± 7.5	29.3 ± 1.4	30.5 ± 1.4	30.6 ± 1.1
*M. integrifolia*	97.9 ± 5.6	96.4 ± 5.9	95.8 ± 6.2	31.8 ± 2.5	32.6 ± 2.5	32.5 ± 2.3
*M. lancifolia*	97.9 ± 6.3	96.9 ± 6.1	96.5 ± 6.1	30.4 ± 2.3	31.5 ± 2.3	31.6 ± 2.1
*M. punicea*	102.0 ± 1.4	101.2 ± 1.7	100.5 ± 1.8	33.5 ± 1.6	34.0 ± 1.4	34.0 ± 1.4
*M. quintuplinervia*	102.0 ± 2.7	98.6 ± 2.5	98.2 ± 2.2	34.2 ± 2.1	34.9 ± 1.7	35.1 ± 1.5
*M. racemosa*	97.7 ± 4.7	95.4 ± 5.6	94.4 ± 5.6	31.4 ± 2.4	31.9 ± 2.4	31.7 ± 2.3

Looking at the predicted shifts in suitable habitat by species basis highlighted the following: Suitable climate space for all species was predicted to shift northward in latitude; whereby, the current range of *M. racemosa* is closest to the future predicted suitable range (0.23°–0.51°) and furthest for *M. lancifolia* (0.53°–1.19°). In addition, all species were predicted to have to shift upwards in elevation to track their current climate; whereby, the discrepancy between current and predicted future elevation is lowest for *M. punicea* (131.12–454.56 m) and highest for *M. quintuplinervia* (399.15–794.74 m). In addition, for the majority of the species suitable climate space is projected to shift westwards in longitude, with the extremes ranging from 0.30° (*M. quintuplinervia*) to 4.79° (*M. impedita*), whereas only *M. impedita* showed a longitudinal increase of 0.70° in one model scenario (HE RCP 8.5). If the current climate data are simplistically taken to represent conditions in the year 1975 (mean of current period: 1960–1990), then the predicted mean rate of elevational shifts in suitable habitat for all species to 2070 would be 33.27 m (17.93 m for *M. punicea* to 54.25 m for *M. quintuplinervia*) per decade for RCP 4.5 and 47.56 m (29.41 m for *M. punicea* to 68.15 m for *M. quintuplinervia*) per decade for RCP 8.5 (Table 2, 3, Figures 3, 4; Supporting information Table S3–S6).

Regarding overall changes in the area of suitable climate, *M. punicea *was predicted to experience a loss (by 3.01%–48.97.0%), while the area of suitable habitat for *M. racemosa* was predicted to expand (by 23.91%–49.30%). The direction of these predictions (reduction and expansion) was consistent across all six models. For the other five species, the models produced conflicting results; whereby for *M. horridula*, *M. integrifolia* and *M. lancifolia,* there was a projected range contraction in three or four out of the six models, and for *M. impedita* and *M. quintuplinervia,* there was a predicted range contraction in respectively one and two models. Generally speaking, these species are predicted to experience a suitable climate range expansion under the RCP 4.5 scenario, and range contractions or little change under the RCP 8.5 scenario (Table 2, Figures 3, 4; Supporting information Table S3, S5).

## 4 DISCUSSION

### 4.1 Historical shifts in elevation

We found that all seven sampled species of *Meconopsis* that occur in the Himalaya–Hengduan Mountains have increased in mean elevation over the past one hundred years. This result is in line with other studies, which show that plant species in the southeastern Swiss Alps (Walther et al., 2005), South America (Feeley, 2012), and California, USA (Wolf et al., 2016), move to higher elevations and cooler habitats as a result of rising temperatures. A significant upward vegetation shift established using historical data covering two centuries has also been shown for the Chimborazo Volcano in Ecuador (Morueta‐Holme et al., 2015). In the present study, six out of seven species shifted significantly upward in elevation from pre‐1970s to post‐1970s, and although nonsignificant, the mean upward elevation shift for the other one species was still non‐negligible.

There has been a steady increase in global temperature since 1970, and the Himalaya–Hengduan Mountains region has an alarming warming rate of 0.6°C per decade, which is considerably higher than the global average (IPCC, 2014; Shrestha, Gautam, & Bawa, 2012). Species of *Meconopsis* are perennial herbs that mainly occur in alpine or subnival habitats, which may be more sensitive to the climate warming and subsequent upward shifts to cooler habitats. Our results contribute to a growing literature base that increasingly suggests that the process of tracking suitable climatic niches through dispersal to relatively cooler habitats may be a ubiquitous response of alpine plant species to climate change at local, regional, and global scales.

### 4.2 Projections in distributional shifts

For all seven species in our study, the modeled predictions of suitable climate consistently indicated that species would need to move to higher elevation and latitudes to track the currently occupied climate niche by the year 2070. These results are in accordance with previous SDM projections across a wide range of species that show a shift either upward or poleward or in both dimensions in suitable climates (Aguirre‐Gutiérrez, van Treuren, Hoekstra, van Hintum, & Vaclavik, 2017; He, Wang, Li, & Yi, 2016; Poudel et al., 2014). We also found evidence for westward shifts in suitable climate. This is attributable to the unique geography of the Himalaya–Hengduan Mountains region where elevation gradually ascends from east to west and species track high‐elevation and high‐latitude habitats that also shift westward in longitude (Liang et al., 2018).

The future projections consistently suggested that the area of suitable habitat will decrease for *M. punicea* and increase for *M. racemosa*. For the other five species (*M. horridula*, *M. impedita*, *M. integrifolia*, *M. lancifolia*, and *M. quintuplinervia*), the area of suitable habitat may either decrease or increase depending on the model and/or scenario, but most of the species were projected to show range expansions in the RCP 4.5 scenario. These results are in line with other vegetation modeling studies in the Himalaya–Hengduan Mountains; whereby, species are often predicted to experience an expansion of suitable climate space upwards and northwards (Liang et al., 2018; You et al., 2018). An increase in suitable habitat does of course not necessarily mean that the species will be able to track it in complex mountain systems. *M. punicea*, the species that was projected to show range contraction among all the models, is a species distributed in relatively high latitude in this region. The species was projected to lose area in the east and southwest margin of the distribution range, and the new potential habitat in the northwest is limited.

### 4.3 Conservation implications in the future

The fact that the seven *Meconopsis* species analyzed here may have experienced an upward shift of elevation suggests that the species are tracking suitable climate space and that future climate predictions are relevant for predicting future range dynamics for these species. Our models consistently showed that suitable climate space will continue to move upwards and northwards. The projected average decadal shifts in suitable climate space are similar to or less than the average decadal upward elevation shift established using historical records (33.2 and 47.6 m per decade under the RCP 4.5 and RCP 8.5 scenarios, respectively, compared to 56.9 m per decade in the historical records). However, whether or not the species will be able to track this suitable space depends on a wide range of factors not being taken into account here—including the complexity of the terrain, dispersal ability, lifespan, climate fluctuations, nonlinear changes, and land use changes (Hulber et al., 2016; Kremer et al., 2012; Pearson, 2006; Thuiller, 2004).

More generally, any broad‐scale climate change threat analysis based on species distribution modeling should be interpreted with great care and accompanied by regular field monitoring. The complex topography and diverse ecological niches that comprise the alpine regions may mitigate potential climate change impacts by providing adequate microhabitats for some species. The species may also have larger climatic tolerances than observed. On the other hand, shifts or fragmentation in suitable climate space, albeit expanding, may still lead to a decline in genetic adaptive variation and population fitness. For this particular study, two potential limitations need to be taken into account: First, most of the pre‐1970s records have an approximate accuracy of 50 or 100 m, whereas post‐1970s records have a higher accuracy; second, there may be a collection bias inherent to the historical elevation records in that remote alpine region over 4,000 m in the Himalaya–Hengduan Mountains may have been less accessible pre‐1970s, which may account for some of the observed elevation shifts in botanical records. Thus, our estimates of historical range shifts may be on the higher side.

Notwithstanding these potential limitations, our results suggest that *Meconopsis* will be impacted by climate change and that the impact will differ for different species. While there may be sufficient space by 2070 with climatic conditions equivalent to those currently experienced by species such as *M. racemosa *and *M. impedita* (currently have a more southerly and easterly range), other species distributed in relatively high latitudes (particularly *M. punicea*, *M. lancifolia*, and *M. integrifolia*) may experience contractions in future suitable habitat. In the absence of any other information, species such as this that are already at their range limits may merit particular consideration in the development of conservation and prioritization strategies.

## CONFLICT OF INTEREST

None declared.

## AUTHOR CONTRIBUTIONS

D.Z.L. and L.M.G. designed the study; X.H. and L.M.G. collected the data; X.H. performed data analysis and generated the graphs; X.H., L.M.G. K.S.B., and A.A. wrote the manuscript; and X.H., K.S.B., L.M.G., X.F.Y., A.A., and D.Z.L. revised the manuscript. All authors read and approved the final manuscript.

## Data Availability

GPS data and elevational records for species in this study are available in Dryad Digital Repository (https://doi.org/10.5061/dryad.d5872s4).
