# Supplementary material for: Upward elevation and northwest range shifts for alpine Meconopsis species in the Himalaya–Hengduan Mountains region
Source: Ecol Evol. 2019 Mar 11;9(7):4055–64. doi: 10.1002/ece3.5034 (PMC6467849; doi:10.1002/ece3.5034)
Supplement: Supplementary file 1 [file ECE3-9-4055-s001.docx]

**APPENDIX**

**Table S1**: Number of original specimens and occurrences used after filtering for model projections of the seven *Meconopsis* species in the Himalaya-Hengduan Mountains.

| Species | Original specimens | Occurrence number |
| --- | --- | --- |
| *M. horridula* | 526 | 252 |
| *M. impedita* | 147 | 19 |
| *M. integrifolia* | 807 | 107 |
| *M. lancifolia* | 162 | 37 |
| *M. punicea* | 303 | 103 |
| *M. quintuplinervia* | 336 | 111 |
| *M. racemosa* | 630 | 164 |

**Table S2:** Correlation matrix of 19 climatic variables used to project range shifts of *Meconopsis* species in the Himalaya-Hengduan Mountains. Values in bold are those with correlation coefficients above 0.90.

|  | Bio1 | Bio2 | Bio3 | Bio4 | Bio5 | Bio6 | Bio7 | Bio8 | Bio9 | Bio10 | Bio11 | Bio12 | Bio13 | Bio14 | Bio15 | Bio16 | Bio17 | Bio18 | Bio19 |
| --- | --- | --- | --- | --- | --- | --- | --- | --- | --- | --- | --- | --- | --- | --- | --- | --- | --- | --- | --- |
| Bio1 | **1.00** | -0.55 | 0.13 | -0.53 | **0.93** | **0.97** | -0.54 | **0.96** | **0.96** | **0.96** | **0.98** | 0.71 | 0.72 | 0.36 | -0.05 | 0.72 | 0.44 | 0.49 | 0.42 |
| Bio2 | -0.55 | **1.00** | 0.23 | 0.48 | -0.36 | -0.67 | 0.72 | -0.48 | -0.50 | -0.47 | -0.58 | -0.70 | -0.55 | -0.73 | 0.57 | -0.58 | -0.76 | -0.62 | -0.70 |
| Bio3 | 0.13 | 0.23 | **1.00** | -0.71 | -0.11 | 0.21 | -0.50 | -0.05 | 0.27 | -0.09 | 0.28 | 0.31 | 0.40 | -0.31 | 0.39 | 0.39 | -0.20 | 0.34 | -0.15 |
| Bio4 | -0.53 | 0.48 | -0.71 | **1.00** | -0.20 | -0.69 | **0.94** | -0.30 | -0.65 | -0.29 | -0.70 | -0.75 | -0.76 | -0.22 | -0.05 | -0.77 | -0.34 | -0.65 | -0.35 |
| Bio5 | **0.93** | -0.36 | -0.11 | -0.20 | **1.00** | 0.82 | -0.20 | **0.96** | 0.85 | **0.99** | 0.83 | 0.46 | 0.50 | 0.25 | 0.02 | 0.50 | 0.30 | 0.21 | 0.28 |
| Bio6 | **0.97** | -0.67 | 0.21 | -0.69 | 0.82 | **1.00** | -0.72 | 0.88 | **0.96** | 0.88 | **0.99** | 0.80 | 0.79 | 0.43 | -0.10 | 0.80 | 0.53 | 0.59 | 0.51 |
| Bio7 | -0.54 | 0.72 | -0.50 | **0.94** | -0.20 | -0.72 | **1.00** | -0.34 | -0.62 | -0.32 | -0.69 | -0.82 | -0.75 | -0.45 | 0.20 | -0.77 | -0.54 | -0.76 | -0.53 |
| Bio8 | **0.96** | -0.48 | -0.05 | -0.30 | **0.96** | 0.88 | -0.34 | **1.00** | 0.87 | **0.99** | 0.88 | 0.58 | 0.58 | 0.32 | -0.09 | 0.58 | 0.37 | 0.39 | 0.33 |
| Bio9 | **0.96** | -0.50 | 0.27 | -0.65 | 0.85 | **0.96** | -0.62 | 0.87 | **1.00** | 0.88 | **0.98** | 0.72 | 0.76 | 0.31 | 0.05 | 0.75 | 0.41 | 0.48 | 0.43 |
| Bio10 | **0.96** | -0.47 | -0.09 | -0.29 | **0.99** | 0.88 | -0.32 | **0.99** | 0.88 | **1.00** | 0.88 | 0.56 | 0.58 | 0.34 | -0.07 | 0.57 | 0.39 | 0.33 | 0.36 |
| Bio11 | **0.98** | -0.58 | 0.28 | -0.70 | 0.83 | **0.99** | -0.69 | 0.88 | **0.98** | 0.88 | **1.00** | 0.79 | 0.80 | 0.36 | -0.03 | 0.80 | 0.46 | 0.57 | 0.44 |
| Bio12 | 0.71 | -0.70 | 0.31 | -0.75 | 0.46 | 0.80 | -0.82 | 0.58 | 0.72 | 0.56 | 0.79 | **1.00** | **0.95** | 0.49 | -0.19 | **0.97** | 0.59 | 0.90 | 0.56 |
| Bio13 | 0.72 | -0.55 | 0.40 | -0.76 | 0.50 | 0.79 | -0.75 | 0.58 | 0.76 | 0.58 | 0.80 | **0.95** | **1.00** | 0.30 | 0.06 | **1.00** | 0.43 | 0.83 | 0.43 |
| Bio14 | 0.36 | -0.73 | -0.31 | -0.22 | 0.25 | 0.43 | -0.45 | 0.32 | 0.31 | 0.34 | 0.36 | 0.49 | 0.30 | **1.00** | -0.62 | 0.32 | **0.96** | 0.42 | 0.89 |
| Bio15 | -0.05 | 0.57 | 0.39 | -0.05 | 0.02 | -0.10 | 0.20 | -0.09 | 0.05 | -0.07 | -0.03 | -0.19 | 0.06 | -0.62 | **1.00** | 0.02 | -0.58 | -0.26 | -0.52 |
| Bio16 | 0.72 | -0.58 | 0.39 | -0.77 | 0.50 | 0.80 | -0.77 | 0.58 | 0.75 | 0.57 | 0.80 | **0.97** | **1.00** | 0.32 | 0.02 | **1.00** | 0.44 | 0.85 | 0.43 |
| Bio17 | 0.44 | -0.76 | -0.20 | -0.34 | 0.30 | 0.53 | -0.54 | 0.37 | 0.41 | 0.39 | 0.46 | 0.59 | 0.43 | **0.96** | -0.58 | 0.44 | **1.00** | 0.50 | **0.96** |
| Bio18 | 0.49 | -0.62 | 0.34 | -0.65 | 0.21 | 0.59 | -0.76 | 0.39 | 0.48 | 0.33 | 0.57 | 0.90 | 0.83 | 0.42 | -0.26 | 0.85 | 0.50 | **1.00** | 0.45 |
| Bio19 | 0.42 | -0.70 | -0.15 | -0.35 | 0.28 | 0.51 | -0.53 | 0.33 | 0.43 | 0.36 | 0.44 | 0.56 | 0.43 | 0.89 | -0.52 | 0.43 | **0.96** | 0.45 | **1.00** |

**Table S3:** RCP 4.5 and RCP 8.5 scenarios of BCC-CSM1-1 (BC) model projections for the average distribution in elevation (m ± standard deviation), the range size (km^2^) and the proportion of range size shift (%) between the current time period and the year 2070 for the seven *Meconopsis* species in the Himalaya-Hengduan Mountains.

| Species | Elevation | | | Range size (Proportion of range size shift) | | | |
| --- | --- | --- | --- | --- | --- | --- | --- |
|  | Current | 2070 RCP 4.5 | 2070 RCP 8.5 | | Current | 2070 RCP 4.5 (%) | 2070 RCP 8.5 (%) |
| *M. horridula* | 4461.9 ± 562.2 | 4614.1 ± 561.3 | 4709.0 ± 482.3 | | 977160.8 | 1060506.7 (8.5) | 859934.3 (-12.0) |
| *M. impedita* | 4155.3 ± 554.3 | 4474.7 ± 564.8 | 4716.3 ± 562.8 | | 133966.1 | 173728.7 (29.7) | 142199.5 (6.1) |
| *M. integrifolia* | 4102.4 ± 660.4 | 4339.8 ± 574.6 | 4443.0 ± 539.5 | | 685546.6 | 748331.4 (9.2) | 622967.4 (-9.1) |
| *M. lancifolia* | 3739.2 ± 936.1 | 4077.1 ± 799.1 | 4265.2 ± 702.5 | | 764798.1 | 796330.1 (4.1) | 714888.3 (-6.5) |
| *M. punicea* | 3699.2 ± 544.9 | 3830.3 ± 470.9 | 3879.8 ± 460.9 | | 224394.0 | 205676.6 (-8.3) | 210889.8 (-6.0) |
| *M. quintuplinervia* | 3602.3 ± 642.1 | 4001.5 ± 528.9 | 4130.8 ± 456.3 | | 255035.0 | 289720.5 (13.6) | 276651.7 (8.5) |
| *M. racemosa* | 4133.9 ± 557.3 | 4372.0 ± 562.8 | 4498.8 ± 510.7 | | 679801.5 | 879991.3 (29.4) | 842341.2 (23.9) |

**Table S4**: RCP 4.5 and RCP 8.5 scenarios of BCC-CSM1-1 (BC) model projections for the average distribution in longitude (°) and latitude (°; ± standard deviation) between the current time period and the year 2070 for the seven *Meconopsis* species in the Himalaya-Hengduan Mountains.

| Species | Longitude | | | Latitude | | | |
| --- | --- | --- | --- | --- | --- | --- | --- |
|  | Current | 2070 RCP 4.5 | 2070 RCP 8.5 | | Current | 2070 RCP 4.5 | 2070 RCP 8.5 |
| *M. horridula* | 95.8 ± 4.9 | 94.3 ± 5.8 | 94.3 ± 5.1 | | 32.0 ± 2.4 | 32.2 ± 2.5 | 32.1 ± 2.4 |
| *M. impedita* | 96.7 ± 7.5 | 95.0 ± 8.2 | 91.9 ± 8.6 | | 29.3 ± 1.4 | 29.7 ± 1.2 | 29.6 ± 1.0 |
| *M. integrifolia* | 97.9 ± 5.6 | 97.1 ± 5.6 | 96.7 ± 5.6 | | 31.8 ± 2.5 | 32.2 ± 2.4 | 32.4 ± 2.6 |
| *M. lancifolia* | 97.9 ± 6.3 | 97.0 ± 6.3 | 96.2 ± 6.5 | | 30.4 ± 2.3 | 31.0 ± 2.3 | 30.9 ± 2.1 |
| *M. punicea* | 102.0 ± 1.4 | 101.7 ± 1.4 | 101.6 ± 1.5 | | 33.5 ± 1.6 | 33.6 ± 1.4 | 33.8 ± 1.5 |
| *M. quintuplinervia* | 102.0 ± 2.7 | 100.0 ± 2.7 | 99.7 ± 2.3 | | 34.2 ± 2.1 | 34.6 ± 1.8 | 34.9 ± 1.9 |
| *M. racemosa* | 97.7 ± 4.7 | 96.0 ± 5.8 | 95.6 ± 5.5 | | 31.4 ± 2.4 | 31.6 ± 2.4 | 31.6 ± 2.4 |

**Table S5:** RCP 4.5 and RCP 8.5 scenarios of HadGEM2-ES (HE) model projections for the average distribution in elevation (m ± standard deviation), the range size (km^2^) and the proportion of range size shift (%) between the current time period and the year 2070 for the seven *Meconopsis* species in the Himalaya-Hengduan Mountains.

| Species | Elevation | | | Range size (Proportion of range size shift) | | | |
| --- | --- | --- | --- | --- | --- | --- | --- |
|  | Current | 2070 RCP 4.5 | 2070 RCP 8.5 | | Current | 2070 RCP 4.5 (%) | 2070 RCP 8.5 (%) |
| *M. horridula* | 4461.9 ± 562.2 | 4677.8 ± 520.5 | 4762.6 ± 475.0 | | 977160.8 | 1005150.4 (2.9) | 835876.6 (-14.5) |
| *M. impedita* | 4155.3 ± 554.3 | 4551.6 ± 507.5 | 4646.3 ± 443.1 | | 133966.1 | 152325.9 (13.7) | 93934.1 (-29.9) |
| *M. integrifolia* | 4102.4 ± 660.4 | 4378.8 ± 556.4 | 4506.1 ± 489.7 | | 685546.6 | 670016.7 (-2.3) | 555159.6 (-19.0) |
| *M. lancifolia* | 3739.2 ± 936.1 | 4140.8 ± 725.9 | 4274.9 ± 623.3 | | 764798.1 | 733702.2 (-4.1) | 654277.8 (-14.5) |
| *M. punicea* | 3699.2 ± 544.9 | 3850.8 ± 491.4 | 3902.3 ± 444.5 | | 224394.0 | 217635.7 (-3.0) | 174476.6 (-22.2) |
| *M. quintuplinervia* | 3602.3 ± 642.1 | 4097.2 ± 505.3 | 4221.2 ± 428.5 | | 255035.0 | 263837.8 (3.5) | 155119.6 (-39.2) |
| *M. racemosa* | 4133.9 ± 557.3 | 4437.5 ± 532.2 | 4553.0 ± 489.6 | | 679801.5 | 936025.4 (37.7) | 1003812.9 (47.7) |

**Table S6**: RCP 4.5 and RCP 8.5 scenarios of HadGEM2-ES (HE) model projections for the average distribution in longitude (°) and latitude (°; ± standard deviation) between the Current time period and the year 2070 for the seven *Meconopsis* species in the Himalaya-Hengduan Mountains.

| Species | Longitude | | | Latitude | | | |
| --- | --- | --- | --- | --- | --- | --- | --- |
|  | Current | 2070 RCP 4.5 | 2070 RCP 8.5 | | Current | 2070 RCP 4.5 | 2070 RCP 8.5 |
| *M. horridula* | 95.8 ± 4.9 | 93.8 ± 5.8 | 93.6 ± 5.6 | | 32.0 ± 2.4 | 32.3 ± 2.4 | 32.6 ± 2.4 |
| *M. impedita* | 96.7 ± 7.5 | 95.3 ± 8.1 | 97.4 ± 6.4 | | 29.3 ± 1.4 | 30.0 ± 1.2 | 30.4 ± 1.3 |
| *M. integrifolia* | 97.9 ± 5.6 | 96.7 ± 6.0 | 96.2 ± 6.1 | | 31.8 ± 2.5 | 32.4 ± 2.4 | 32.7 ± 2.5 |
| *M. lancifolia* | 97.9 ± 6.3 | 96.9 ± 6.5 | 97.0 ± 6.1 | | 30.4 ± 2.3 | 31.1 ± 2.3 | 31.5 ± 2.2 |
| *M. punicea* | 102.0 ± 1.4 | 101.4 ± 1.6 | 101.3 ± 1.7 | | 33.5 ± 1.6 | 34.0 ± 1.5 | 34.3 ± 1.5 |
| *M. quintuplinervia* | 102.0 ± 2.7 | 99.4 ± 2.8 | 99.0 ± 2.2 | | 34.2 ± 2.1 | 34.9 ± 1.5 | 35.6 ± 1.7 |
| *M. racemosa* | 97.7 ± 4.7 | 95.4 ± 5.8 | 94.6 ± 5.7 | | 31.4 ± 2.4 | 31.6 ± 2.3 | 31.8 ± 2.3 |


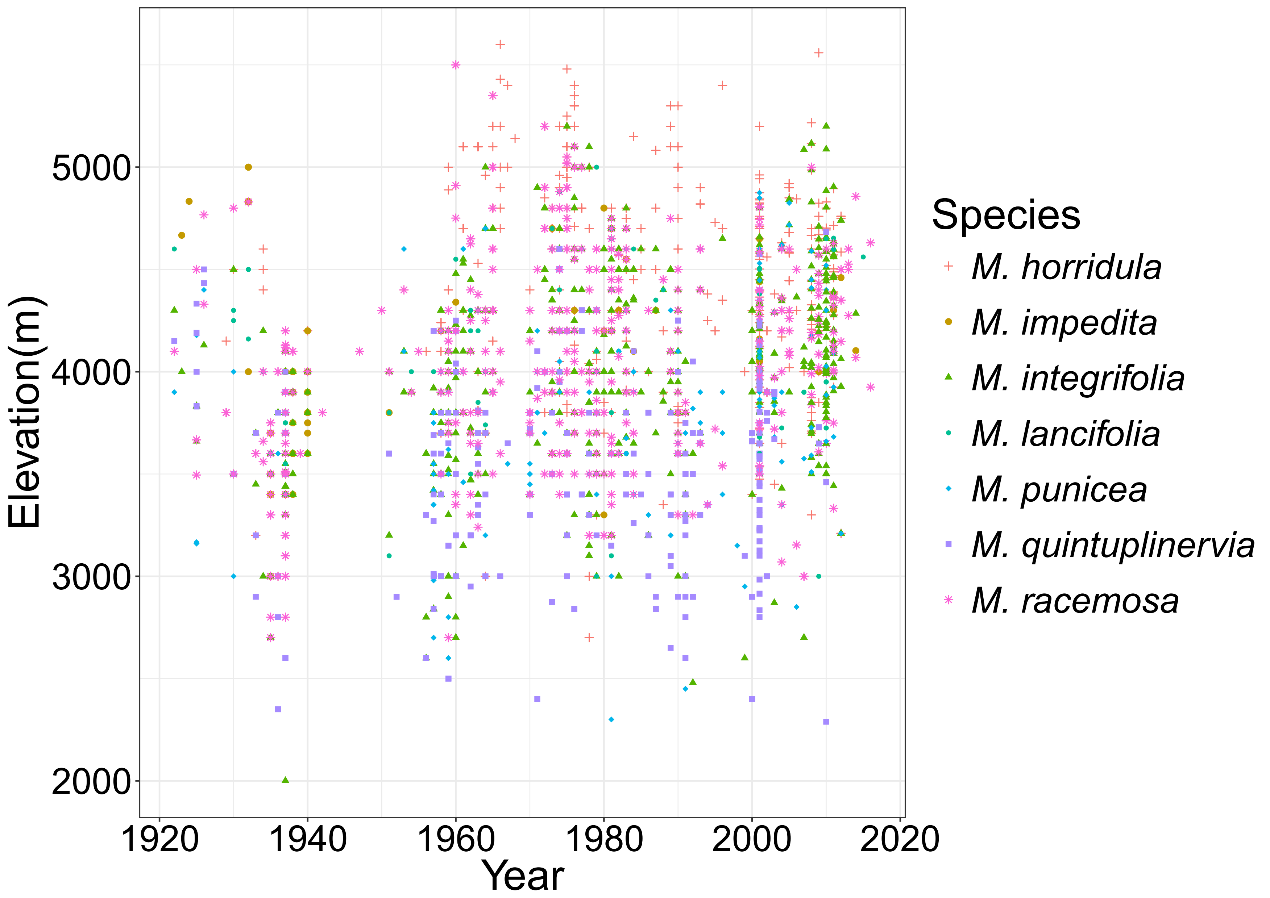


**Figure S1:** Occurrence of elevation records spanning 1922 to 2016 for the seven species of *Meconopsis* in Himalaya-Hengduan Mountains.


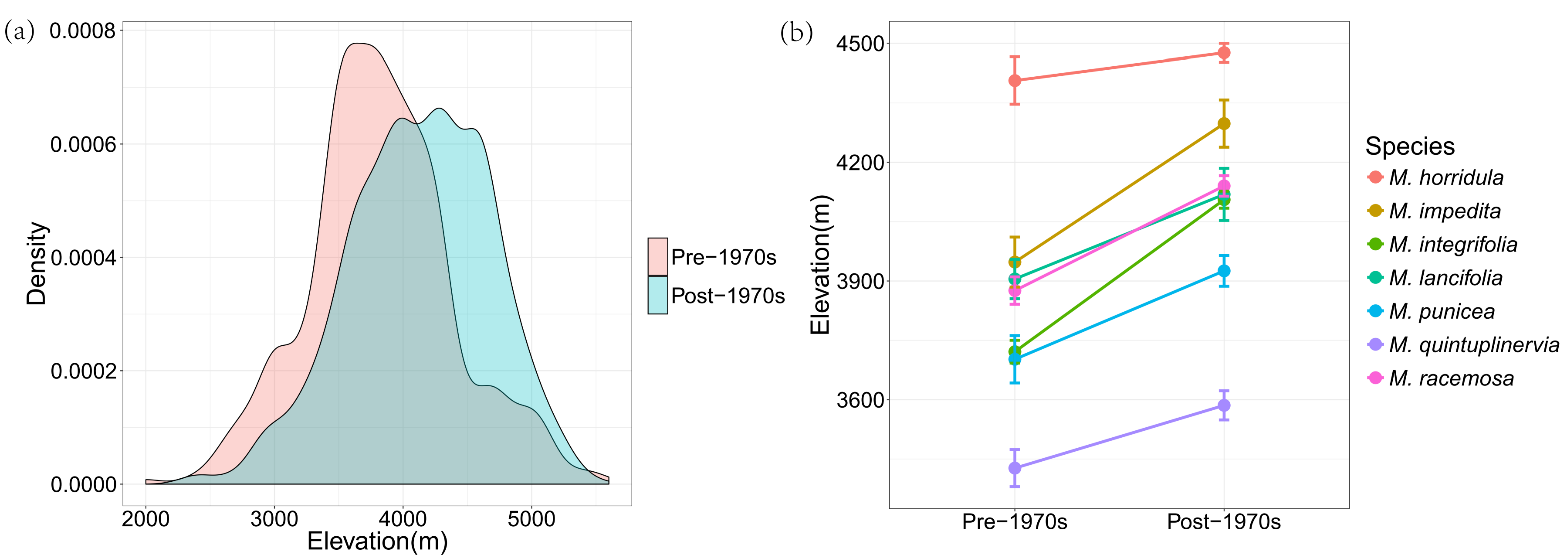


**Figure S2:** Distribution of historical data and comparative analysis of elevational shifts in the Himalaya-Hengduan Mountains. (a) Frequency histogram of elevation records for seven species of *Meconopsis* in the two time periods: pre-1970s (1922-1969) and post-1970s (1970-2016). (b) Mean elevation (m ± standard error) for seven species of *Meconopsis* in the two time periods: pre-1970s (1922-1969) and post-1970s (1970-2016).
